# Supplementary material for: A light tunable differentiation system for the creation and control of consortia in yeast
Source: Nat Commun. 2021 Oct 5;12:5829. doi: 10.1038/s41467-021-26129-7 (PMC8492667; doi:10.1038/s41467-021-26129-7)
Supplement: Supplementary file 2 — Reporting Summary [file 41467_2021_26129_MOESM2_ESM.pdf]

nature portfolio

Corresponding author(s): Gregory Batt & Jakob Ruess

Last updated by author(s): Aug 28, 2021

Reporting Summary

Nature Portfolio wishes to improve the reproducibility of the work that we publish. This form provides structure for consistency and transparency in reporting. For further information on Nature Portfolio policies, see our Editorial Policies and the Editorial Policy Checklist.

Statistics

For all statistical analyses, confirm that the following items are present in the figure legend, table legend, main text, or Methods section.

n/a	Confirmed	
☐	☒	The exact sample size (*n*) for each experimental group/condition, given as a discrete number and unit of measurement
☒	☐	A statement on whether measurements were taken from distinct samples or whether the same sample was measured repeatedly
☐	☒	The statistical test(s) used AND whether they are one- or two-sided<br>*Only common tests should be described solely by name; describe more complex techniques in the Methods section.*
☒	☐	A description of all covariates tested
☒	☐	A description of any assumptions or corrections, such as tests of normality and adjustment for multiple comparisons
☐	☒	A full description of the statistical parameters including central tendency (e.g. means) or other basic estimates (e.g. regression coefficient) AND variation (e.g. standard deviation) or associated estimates of uncertainty (e.g. confidence intervals)
☐	☒	For null hypothesis testing, the test statistic (e.g. *F*, *t*, *r*) with confidence intervals, effect sizes, degrees of freedom and *P* value noted<br>*Give P values as exact values whenever suitable.*
☒	☐	For Bayesian analysis, information on the choice of priors and Markov chain Monte Carlo settings
☒	☐	For hierarchical and complex designs, identification of the appropriate level for tests and full reporting of outcomes
☒	☐	Estimates of effect sizes (e.g. Cohen's *d*, Pearson's *r*), indicating how they were calculated

Our web collection on statistics for biologists contains articles on many of the points above.

Software and code

Policy information about availability of computer code

Data collection	Cytometry data was acquired with GuavaSoft InCyte software (version 3.3) according to manufacturer's recommendations. Optical density data was acquired using ReacSight, a generic software framework for bioreactor arrays, described elsewhere (BioRxiv, doi: 10.1101/2020.12.27.424467).<br>Live cell imaging and pattern formation experiments utilized custom microscopy software, MicroMator, described elsewhere (BioRxiv, doi: 10.1101/2021.03.12.435206).<br>Mathematical models were simulated in Python 3 with the help of SciPy 1.4.0.
Data analysis	Bioreactor data (including cytometry) was parsed and analyzed in Python using Jupyter notebooks.<br>Microscopy images were segmented using Segmator, described with the MicroMator tool, and all subsequent analysis was carried out in Python. Images shown in the manuscript were processed using FIJI (version ImageJ 1.52i).<br>Jupyter notebooks that allow for the reproduction of analysis or raw data and the generation of figures appearing in the article can be found in the GitLab repository. https://gitlab.inria.fr/InBio/Public/YeastOptogeneticDifferentiation_YODA

For manuscripts utilizing custom algorithms or software that are central to the research but not yet described in published literature, software must be made available to editors and reviewers. We strongly encourage code deposition in a community repository (e.g. GitHub). See the Nature Portfolio guidelines for submitting code & software for further information.

Data

Policy information about availability of data

All manuscripts must include a data availability statement. This statement should provide the following information, where applicable:

- Accession codes, unique identifiers, or web links for publicly available datasets
- A description of any restrictions on data availability
- For clinical datasets or third party data, please ensure that the statement adheres to our policy

Raw cytometry and OD data generated in this study and processed microscopy data have been deposited on Zenodo under the ascension code 4923833 (https://doi.org/10.5281/zenodo.4923833).

Field-specific reporting

Please select the one below that is the best fit for your research. If you are not sure, read the appropriate sections before making your selection.

☒ Life sciences ☐ Behavioural & social sciences ☐ Ecological, evolutionary & environmental sciences

For a reference copy of the document with all sections, see nature.com/documents/nr-reporting-summary-flat.pdf

Life sciences study design

All studies must disclose on these points even when the disclosure is negative.

Sample size	For each timepoint of bioreactor experiments, sample size was determined by cytometry counts. 5000 events were recorded at each timepoint. We were restricted to acquiring 5000 events due to timing constraints imposed by the sampling frequency. This sample size was sufficient for the purposes of control experiments because, statistically, the error in estimation of differentiation fraction was orders of magnitude lower than day-to-day or reactor-to-reactor variability.<br>For the sample size of each experiment we were limited by the number of available bioreactors as well as the duration of experiments.<br>For efficiency and leakage experiments, manual acquisition of timepoints allowed us to acquire 20 replicates with 50000 events for each condition at each timepoint. This was necessary to obtain satisfactorily accurate estimation of the leakage and efficiency.<br>Sample sizes for microscopy experiments were dictated by the constraints of the equipment used to hold cells i.e. number of chambers or wells in the microfluidic plate or µIbidi slide.
Data exclusions	Time series cytometry data was cleaned by removing timepoints that featured cross contamination from other reactors. This cross-contamination was sporadic in nature. The criterion was pre-established.<br>In addition to this, certain timepoints in the two reactor MPC experiments were removed due to malfunctions in OD control. This was necessary as the model predictive control was deployed to operate at predefined ODs (cell density being a parameter in the model). This exclusion is noted in the main text, next to the experiments that featured data exclusion. The criterion was evident, however, not pre-established.<br>Data from one field of view for one replicate of the characterization experiment in the microscope was excluded from data analysis due to lack of cell growth. The criterion was not pre-established.
Replication	All data acquired in the bioreactor platform was reproducible upto a small reactor-to-reactor variability.<br>Efficiency and leakage experiment was performed with 20 replicates. Each of the 20 replicates had a technical replicate to ensure that the time spent inside the cytometer did not influence the results. All replicates were consistent and have been plotted as individual data points.<br>Bioreactor characterization experiments were performed in duplicates (repeated pulses experiments) and triplicates (single pulse experiments) depending on the availability of bioreactors. All replicates were consistent. For repeated pulse experiments, individual data points are shown.<br>Due to differences in the duration of experiments, timeseries data from only the longest set of experiments is shown.<br>GAuDi and multi-species experiments were performed once.<br>Microscopy characterization experiments were performed in duplicates and both replicates were consistent.<br>All four pattern formation experiments were performed twice independently of each other and led to consistent results with an unmistakable pattern emerging in each replicate.
Randomization	Samples were allocated to different groups based on the amount of light they received.<br>There was no randomization for experiment design, data collection or data analysis because isogenic yeast were used for all the experiments and therefore no randomization was deemed necessary to control for genomic differences.
Blinding	Blinding does not apply to our study as both data acquisition and analysis were automated. We note, however, that for the efficiency and leakage experiment data was acquired in a semi-automated fashion by manually diluting the cell culture and manually placing the 96-well plate in the cytometer.

Reporting for specific materials, systems and methods

We require information from authors about some types of materials, experimental systems and methods used in many studies. Here, indicate whether each material, system or method listed is relevant to your study. If you are not sure if a list item applies to your research, read the appropriate section before selecting a response.

Materials & experimental systems

n/a	Involved in the study
☒	☐ Antibodies
☒	☐ Eukaryotic cell lines
☒	☐ Palaeontology and archaeology
☒	☐ Animals and other organisms
☒	☐ Human research participants
☒	☐ Clinical data
☒	☐ Dual use research of concern

Methods

n/a	Involved in the study
☒	☐ ChIP-seq
☐	☒ Flow cytometry
☒	☐ MRI-based neuroimaging

Flow Cytometry

Plots

Confirm that:

☒ The axis labels state the marker and fluorochrome used (e.g. CD4-FITC).

☒ The axis scales are clearly visible. Include numbers along axes only for bottom left plot of group (a 'group' is an analysis of identical markers).

☒ All plots are contour plots with outliers or pseudocolor plots.

☒ A numerical value for number of cells or percentage (with statistics) is provided.

Methodology

Sample preparation	Cells were cultured continuously in exponential phase between OD 0.4 and OD 0.6 using a bioreactor array. Prior to cytometry measurement, the sample was diluted 20 times in PBS.
Instrument	We used a benchtop flow cytometer Guava EasyCyte HT BGV (0500-4030)
Software	GuavaSoft InCyte (version 3.3) software recommended by the manufacturer was used for data collection. No compensation was used during acquisition. Raw cytometry data was parsed and analyzed in Python. Jupyter notebooks are available online and allow reproduction data analysis and the generation of figures (https://gitlab.inria.fr/InBio/Public/YeastOptogeneticDifferentiation_YODA).
Cell population abundance	For all experiments, except those with the GAuDi strain, dead cells were less than 1% of the gated singlets and were excluded from analysis. For the GAuDi strains, the fraction of dead cells was variable and was explicitly described in the corresponding plots.<br>Cell population abundance was calculated by applying thresholds in relevant fluorescence channels as described in the supplementary information text.
Gating strategy	Gating was done using kernel density based methods as described in the ReacSight manuscript (see above). Size gating was performed on FSCH vs SSCH to remove debris and cells with aberrant phenotype. Singlets were selected based on deviation from linearity in Forward Scatter Height (FSC-H) vs. Forward Scatter Area (FSC-A). Cells were scored and a threshold was defined above which cells were classified as doublets and removed from analysis. For size gating, 2D kernel density estimates were obtained using SciPy gaussian kde package on Forward Scatter (FSC-H) vs. Side Scatter (SSC-H) and regions of density lower than a threshold were removed. The two thresholds were kept constant for all measurements except those made with the GAuDi strain. In the latter case, thresholds were increased to include the entire population. This leniency was warranted because of considerable changes in the side scatter in growth arrested cells.

☒ Tick this box to confirm that a figure exemplifying the gating strategy is provided in the Supplementary Information.
