# Supplementary material for: A light tunable differentiation system for the creation and control of consortia in yeast
Source: Nat Commun. 2021 Oct 5;12:5829. doi: 10.1038/s41467-021-26129-7 (PMC8492667; doi:10.1038/s41467-021-26129-7)
Supplement: Supplementary file 3 — Description of Additional Supplementary Files [file 41467_2021_26129_MOESM3_ESM.pdf]

Title: Supplementary Data 1.
Description: Sequences of plasmids and backbones used in this study.

Title: Supplementary Movie 1.
Description: Differentiation behaviour and cell growth of cells carrying the original differentiation system under the microscope in a microfluidic plate. Induction started at t=0 with 800ms pulses. Cyan represents mCerulean fluorescence acquired in the CFP channel and yellow represents mNeonGreen fluorescence acquired in the YFP channel (Table S2). The video shows a merge of brightfield, CFP, and YFP channels.

Title: Supplementary Movie 2.
Description: Differentiation behaviour and growth arrest of GAuDi02 cells under the microscope in a microfluidic plate. Induction started at t=0 with 800ms pulses. Cyan represents mCerulean fluorescence acquired in the CFP channel, yellow represents mNeonGreen fluorescence acquired in the YFP channel, and magenta represents mScarlet-I fluorescence acquired in the Rhodamine channel (Table S2). The video shows a merge of brightfield, CFP, YFP, and RHOD channels. Images were cropped to increase resolution while retaining all the cells in the field of view.
